# Contrasting HIV prevalence trends among young women and men in Zambia in the past 12 years: data from demographic and health surveys 2002–2014

**DOI:** 10.1186/s12879-019-4059-3

**Published:** 2019-05-17

**Authors:** Chola Nakazwe, Charles Michelo, Ingvild F. Sandøy, Knut Fylkesnes

**Affiliations:** 1 Centre for International Health, University of Bergen, Bergen, Norway; 2 School of Public Health, University of Zambia, Lusaka, Zambia; 3 Central Statistical Office, Lusaka, Zambia; 4 Department of Global Public Health and Primary Care, Centre for Intervention Science in Maternal and Child Health, University of Bergen, Bergen, Norway

**Keywords:** HIV prevalence - AIDS- young people - Zambia

## Abstract

**Background:**

The HIV epidemic remains a concern on the global health agenda, despite progress made in reducing incidence. Investigation of trends among young people is important for monitoring HIV incidence and informing programming. The study examined geographical and sub-population differences in HIV prevalence trends among young people aged 15–24 years in Zambia.

**Methods:**

This study analysed data from Zambia Demographic and Health Surveys (ZDHSs) that were conducted in 2001–2, 2007, and 2013–14. A two-stage cluster stratified sampling procedure was used to select samples of 8050, 7969, and 18,052 for the three surveys, respectively. Young people (15–24 years) with known HIV status were selected for analysis. The outcome variable was HIV status. Log binomial regression analysis of generalised linear models was used to test for trends.

**Results:**

Overall HIV prevalence declined over the period 2001–2 to 2013–14 among women and men aged 15–49 years (17.8 and 12.9% to 15.1 and 11.3%, respectively). There was, however, an increase in HIV prevalence among urban young men over this period, from 3.7% in 2001–2 to 7.3% in 2013–14 (aRR 2.17, 95% CI 0.99˗4.75), and, in rural areas, from 2.6 to 3.6% (aRR 1.46, 95% CI 0.78˗2.75). In contrast, HIV prevalence among women declined over the same period of time. In urban areas, HIV prevalence among women declined from 15.2 to 10.7% (aRR 0.66, 95% CI 0.53˗0.93), while in rural areas it declined from 8.2 to 4.8% (aRR 0.41, 95% CI 0.59˗0.85). In addition, there was a narrowing gender gap in terms of HIV infection, as the prevalence ratio of females to males declined from 4.2 and 3.1 to 1.5 and 1.3, in urban and rural areas, respectively.

**Conclusions:**

The increase in HIV prevalence among urban young men over the past 12 years, contrasting declining trends among young women in both urban and rural populations, suggests differential effects of prevention efforts. Furthermore, findings that Zambia’s overall national HIV prevalence decline masks some striking sex and rural/urban differentials, indicate the need for reconsidering the prevention efforts for young urban men.

## Background

The HIV epidemic remains a major concern on the global health agenda, despite the progress made in reducing incidence and improving access to antiretroviral treatment. The focus of the post-2015 era has been nothing less than the end of the AIDS epidemic by 2030 through the implementation of an ambitious Joint United Nations Programme on HIV/AIDS (UNAIDS) 90–90-90 treatment target [1]. UNAIDS currently estimates that 36.7 million people are living with HIV infection. In sub-Saharan Africa, Eastern and Southern Africa are the hardest hit, with more than half of people living with HIV residing in this region, despite the fact that only 6.2% of the world’s population resides in this region [2]. The HIV incidence in sub-Saharan Africa peaked during the mid-1990s and substantially declined during the subsequent 10–15 years [3]. However, since 2010, this consistent declining pattern among adults seems to have halted, with only marginal overall declines apparent in Eastern and Southern Africa and with some countries experiencing an increase in incidence [3, 4].

Monitoring HIV prevalence, patterns, and trends is important for efficient planning, resource allocation, and informing programmes. At the global level, antenatal clinic (ANC)-based surveillance has formed the basis for the measurement of prevalence (both at local and national levels), and prevalence change among young women, which has been used as a proxy for change in incidence. Estimates of trends in incidence and mortality are mathematically modelled from ANC and other available data by the UNAIDS expert group. The UNAIDS/WHO Spectrum AIDS Impact Model is the most commonly used method for deriving HIV incidence [5, 6]. However, ANC data has some inherent biases, including the exclusion of men, non-pregnant women, and sexually inactive persons. Therefore, population-based surveys that provide HIV prevalence data have become increasingly important. Nationally representative household surveys that are repeated periodically are used to monitor prevalence and estimate incidence in age-specific population groups. Specifically, prevalence trends among young people at subnational levels are an indicator of incidence within the general population [7]. In recent years, countries in sub-Saharan Africa have included HIV incidence assays and testing algorithms in population based surveys to measure HIV incidence and monitor HIV impact in accordance with the UNAIDS/WHO recommendation [8]. Zambia implemented the population-based HIV impact assessment survey (ZAMPHIA) in 2016, the first survey to measure national incidence [9].

Zambia is one of 15 countries in the world that have the highest HIV disease burden. It is the leading cause of death among adults aged 15 years and older in Zambia with estimates of about 20% of adult deaths attributed to HIV [10]. An estimated 6.6% of young people are HIV positive and prevalence is higher among young women (8%) than men (5%). Annual HIV incidence among persons 15–24 years is 57 new infections per 10,000 uninfected persons per year.

Monitoring HIV prevalence and associated risk factors in Zambia is based on a comprehensive ANC-based surveillance system, population-based surveys in selected communities, and nationally representative population-based surveys. ANC-based data covering the period of 1994–2011 showed declining overall prevalence trends among young women; however, these trend patterns differed significantly according to place and educational attainment [11–13]. Similarly, a series of cross-sectional population-based surveys conducted in select communities in 1995, 1999, and 2003 showed marked declines in prevalence both among urban and rural men and women, with declines being associated with changes in behaviour and strongly skewed towards the higher educated [14–16]. Furthermore, population-based survey systems have been used to validate the representativeness of ANC-based data at various levels. Similar prevalence estimates were found at national level using ZDHS 2001–2 as a comparison [17], and at local level, using data from selected communities [11]. With respect to trends in prevalence among young people, the ANC-based data somewhat underestimated the declines in comparison with the population-based surveys that were conducted in the same local settings [18].

HIV prevalence among young people (15–24 years) in repeated cross-sectional surveys can be used as a good proxy to monitor changes in incidence over time because the prevalence in this age group is only marginally affected by mortality or treatment [19, 20]. They are also at a stage when sexual and reproductive activity, including risky sexual behaviour, usually begins. With the high burden of HIV infection in Zambia, more detailed analyses of changes in prevalence among young people in rural/urban sub-populations and by sex than what is provided in the published DHS reports, can provide useful information for evaluating epidemic control efforts. This article examines geographic and sub-population differences in HIV prevalence trends among young people from three rounds of the Zambia Demographic and Health Surveys (2001–2, 2007, and 2013–14).

## Methods

### Settings

Zambia is located in Southern Africa with a total land area of 752,612 sq. km. The current population is estimated at 13 million people with an annual growth rate of 2.8. More than a third (39%) of the population live in urban areas. About two-thirds of the population is below the age of 25 years with 22% being between 15 and 24 years [21]. The estimated HIV prevalence among those aged 15–49 years is 13.3 and 6.6% among young people aged 15–24 years [22].

### Data collection

The Zambia Demographic and Health Survey (ZDHS) is a cross-sectional nationally representative population-based survey, which has included an HIV testing module since 2001–2. A two-stage stratified cluster sampling procedure was followed in each of the ZDHS rounds, based on the 2000 population census frame for the 2001–2 and 2007 ZDHS and the 2010 population census frame for the 2013/14 ZDHS. Stratification was achieved by separating each province into rural and urban areas. Therefore, the nine provinces gave 18 sampling strata in 2001–2 and 2007, and in 2013–14, when a new province was partitioned, there were 20 sampling strata (strata are non-overlapping subgroups from which households or populations are sampled). The samples for the 2001–2 and 2007 ZDHS were designed to provide estimates of population and health indicators at the national and provincial levels. The 2013–14 ZDHS sample was designed to provide estimates at the national level, provincial level, and rural and urban areas within provinces. At the first stage, primary sampling units or clusters (EAs) were selected from the strata with probability proportional to size. An Enumeration Area (EA) is a convenient geographic area with an average size of 90 and 130 households in rural and urban areas, respectively. All households in selected clusters were listed for the three rounds of ZDHS, and an equal probability systematic sampling of households was done to obtain target sample sizes of 8050, 7969, and 18,052 households, respectively. All women 15–49 years and men 15–59 years were eligible to participate in the surveys, except in the 2001–2 survey where men were only eligible in one-third of the selected households. Eligible women and men living in the households selected for the survey were asked to consent to the syphilis and HIV testing. Young people (aged 15–24 years) accounted for 44% (or 4280) of the sample in 2001–2 and 41% (or 5426 and 12,303) in the two subsequent surveys. In total, 1615, 4235, and 11,571 young people aged 15–24 years with known HIV status (i.e. those who were successfully interviewed and consented to providing a Dried Blood Spot (DBS) or venous blood draw for the HIV testing) were selected for analysis by the three surveys, respectively. The detailed methods of the three surveys are reported in the main Zambia Demographic and Health Survey reports [22–24].

In the ZDHS 2001–2, HIV testing was anonymous and unlinked to socio-demographic variables apart from sex, age, urban-rural residence, and province. Venous blood specimens were collected from consenting participants and dried blood spot (DBS) samples were prepared on filter paper for testing. The DBS samples were screened for HIV antibodies using Wellcozyme HIV 1 & 2 GACELISA. All positive samples and 10% of the negative samples were retested using BIONOR HIV 1 & 2 and discordant specimens were tested with Western Blot. In the 2007 and 2013–14 surveys, blood specimens in the form of Dried Blood pots (DBS) were collected using finger pricking. The protocols for HIV testing for these two surveys allowed for anonymous linking of the HIV results to the socio-demographic data of individuals interviewed. Vironostika HIV Antigen /Antibody Combination Assay (Biomerieux) was used for HIV antibody screening and all positive samples were retested using ELISA Enzygnost HIV Integral II Assay (Dade Behring) for confirmatory testing. For discordant results, specimens were retested using the two tests. If specimens were still discrepant, Western Blot was used as a third confirmatory test. Further details about the testing methodology can be found in the ZDHS reports [22–24].

### Ethics

The Ethical Review Committee at the University of Zambia and the Institutional Review Board of ORC Macro approved the 2001–2 ZDHS. The two subsequent surveys obtained ethical approval from the Tropical Disease and Research Centre (TDRC) ethical committee, the Institutional Review Board of Macro International and the Centres for Disease Control and Prevention (CDC) Atlanta research ethics review board. Participation in the surveys was based on informed and voluntary consent, and separate consent was sought for HIV and syphilis testing. Participants were informed that the survey HIV testing was anonymous. However, a rapid test was offered to participants in the 2013–14 round by nurse and lay counsellors who provided homebased counselling and testing following the national HIV testing algorithm to ascertain HIV infection status for respondents who consented to it. Concurrent HIV testing with Determine™ HIV ½ and Uni-Gold™ was the field testing procedure. For samples reactive (positive) to either rapid test, further consent was sought to conduct a venous blood draw for CD4 count testing, using the PIMA Point of Care CD4 machine. CD4 count results were returned to participants within 24 h. Participants were informed about these procedures in accordance with ethical requirements.

### Statistical analysis

Analysis included all survey participants but with a focus on young people aged 15–24 years. Stratifications was done by sex and urban/rural residence. The data is restricted to three data point periods of the population-based surveys. Data were analysed using STATA version 14.2, and all analyses were weighted to adjust for complex study design. The sample weights also accounted for differences in the non-response by geographic region and sex. Log-binomial regression analysis of the generalised linear model was used to test for trends by determining the statistical significance of the change in prevalence in the period 2001–2 to 2013–14. The analysis also controlled for the potential confounder age, which was adjusted for as a linear effect.

## Results

### Participation

The overall participation rates for the three surveys were similar. In all three surveys, 98% of the households were successfully interviewed. Participation rates were higher among women than men (96, 97, and 91% compared to 91, 91, and 89%, respectively). Details of participation in the 2001/2, 2007, and 2013/14 ZDHS are published in the detailed reports [22–24].

Participation in the HIV testing was closely similar in the first two surveys in 2001–2 and 2007 (72–73% for men and 77–79% for women) and about 10% higher in the last survey (Table 1). Participation was similar for rural men and women but substantially higher among urban women than men. Overall response rates were lower among men and participants residing in urban areas (Table 1). Details of HIV participation in the 2001–2, 2007, and 2013–14 ZDHS are published in the detailed reports [22–24].

**Table 1:** HIV participation rates (percentages) of the 2001/2, 2007, and 2013/14 Zambia Demographic and Health Survey (ZDHS)

Background Variables	2001/2				2007				2013/14			
	Women		Men		Women		Men		Women		Men	
	No.	% Tested	No.	% Tested	No.	% Tested	No.	% Tested	No.	% Tested	No.	% Tested
Age group												
15–19	629	81.1	459	75.2	1665	76.0	1533	72.5	3840	90.8	3617	85.8
20–24	570	80.3	346	83.4	1476	74.9	1170	70.8	3175	91.0	2532	84.8
25–29	455	80.9	361	79.0	1414	78.1	1104	69.5	2891	90.9	2127	84.0
30–34	334	81.5	281	80.5	1071	78.5	1042	72.0	2514	90.4	2091	82.3
35–39	250	84.5	241	87.3	756	78.0	818	72.7	2054	89.5	1858	82.5
40–44	202	81.3	174	80.6	551	78.8	512	76.4	1527	89.6	1534	82.4
45–49	164	78.8	113	76.9	475	79.2	427	73.3	1062	89.5	1101	81.5
50–54	na	na	100	86.8	na	na	319	74.0	na	na	1349	83.3
55–59	na	na	71	74.2	na	na	221	76.5	na	na		
Residence												
Rural	1816	79.3	1604	76.8	4088	77.7	3921	75.8	8852	91.7	8549	87.3
Urban	873	79.5	814	66.5	3320	76.4	3225	67.8	8212	89.1	7660	79.8
Overall Response		79.4		73.3		77.1		72.2		90.4		83.7

### Trends overall

**Fig. 1:**
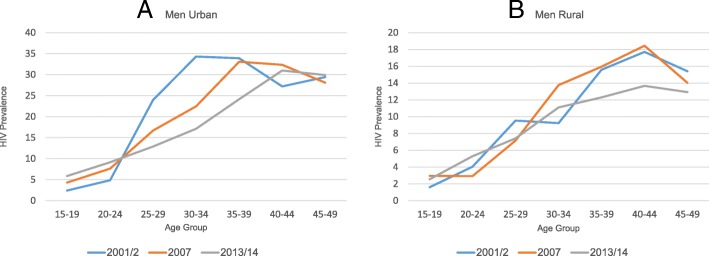
**a**-**b** HIV prevalence by age and rural/urban among young men 15–24 years, 2001–2, 2007, and 2013–14 ZDHS

**Fig. 2:**
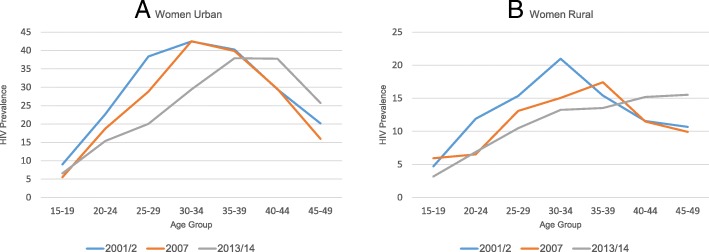
**a**-**b** HIV prevalence by age and rural/urban among young women 15–24 years, 2001–2, 2007, and 2013–14 ZDHS

Overall HIV prevalence declined over the period 2001/2 to 2013/14 among women and men aged 15–49 years (17.8 and 12.9% to 15.1 and 11.3%, respectively). A similar decline was observed in both urban and rural areas (23.1 and 10.8% to 18.2 and 9.1%, respectively) for men and women combined. The decline was steeper among women in urban areas (26.3 to 21.1%) compared to males (8.9 to 8.1%), data not shown. The age-specific patterns of change in HIV prevalence over time among men and women show striking differences, particularly in the urban areas (Figs. 1 and 2). Whereas HIV increased in the youngest two 5-year age groups among young men in urban areas, the prevalence declined substantially in the next three 5-year age groups (25–39 years) and was rather stable among those 40 or older (Fig. 1a). Among rural men, this pattern of change over time was less prominent or partly unstable with the exception of a tendency of declines among those aged 35–49 years (Fig. 1b). Among women, the age-specific prevalence estimates declined in all age groups with the exception of an observed increase in HIV prevalence among those aged 40–49 both in urban and rural areas (Fig. 2).

### Trends among young people

A higher proportion of young women was HIV-infected than young men in the three survey rounds. The sex differentials by rural/urban residence persisted with a higher proportion of young women than young men infected in urban than rural areas (Table 2). HIV prevalence declined in a linear pattern between 2001–2 and 2013–14 among young women from 15.2 to 10.7% (aRR 0.66, 95% CI: 0.53–0.93) and 8.2 to 4.8% (aRR 0.59, 95% CI 0.41–0.85), in urban and rural areas, respectively. Conversely, there was a linear increase in HIV prevalence among young men in both urban and rural populations (3.7 to 7.3% (aRR 2.17, 95% CI: 0.99–4.75) and 2.6 to 3.6% (aRR 1.46, 95% CI 0.78–2.75)), but only the trend among urban men was of statistical significance (Table 2). Among the young people aged 15–19 and 20–24, there was a narrowing gender gap in terms of HIV infection. In 2001–2, the women/men prevalence ratio (15–24 years) was 4.2 and 3.1 in urban and rural areas, respectively. This ratio reduced to 2.0 and 2.1 in urban and rural areas, respectively in 2007, and narrowed further to 1.5 and 1.3 in 2013/14.

**Table 2:** HIV prevalence and age-adjusted risk ratios estimates for ZDHS (2001–2, 2007, and 2013–14) by rural/urban residence, province, and age group among young people (aged 15–24 years)

	Women					Men				
	2001/2	2007	2013/14	2001/2–2013/14	*P*-value	2001/2	2007	2013/14	2001/2–2013/14	P-value
	%	%	%	aOR (CI)		%	%	%	aOR (CI)	
Urban	15.2	11.2	10.7	0.66 (0.53–0.93)**	0.02	3.7	5.7	7.3	2.17 (0.99–4.75)**	**0.05**
Age Group										
15–19	9.0	5.5	6.6	0.73 (0.41–1.30)	0.29	2.4	4.3	5.9	2.52 (0.60–10.62)	0.21
20–24	22.7	18.8	15.4	0.63 (0.41–0.96)**	0.03	4.9	7.6	9.1	1.87 (0.77–5.04)	0.16
Province										
Central	15.9	15.3	9.8	0.50(0.17–1.39)	0.18	4.3	10.4	3.9	0.88 (0.10–7.54)	0.91
Copperbelt	12.3	8.9	10.6	0.82(0.43–1.56)	0.55	2.8	5.2	10.7	4.34 (1.03–18.32)**	0.05
Eastern	0.0	11.1	11.0			0.0	2.9	4.9	–	
Luapula	11.1	10.5	18.3	1.87(0.25–13.81)	0.54	0.0	1.6	9.5	–	
Lusaka	20.8	10.8	9.0	0.38(0.21–0.69)***	0.00	6.0	7.3	4.3	0.75 (0.21–2.66)	0.66
Muchinga	–	–	6.5	–		–	–	6.4	–	
Northern	11.8	8.8	9.4	0.62(0.12–3.10)	0.56	0.0	1.0	6.8	–	
N/Western	5.9	9.4	7.9	1.37(0.18–11.25)	0.77	6.8	0.0	5.3	0.99 (0.14–7.21)	0.99
Southern	21.7	19.7	17.6	0.79(0.27 - 2.30)	0.67	0.0	5.2	8.9	–	
Western	18.2	21.1	19.2	1.36(0.31–6.08)	0.68	16.7	9.4	11.8	0.52 (0.06–4.85)	0.56
Rural	8.2	6.2	4.8	0.59(0.41–0.85)***	0.01	2.6	2.9	3.6	1.46 (0.78–2.75)	0.24
Age Group										
15–19	4.7	5.9	3.2	0.68 (0.38–1.23)	0.20	1.6	3.0	2.5	1.64 (0.69–3.89)	0.26
20–14	11.9	6.5	6.9	0.57(0.36–0.89)***	0.01	4.0	2.9	5.3	1.40 (0.59–3.33)	0.45
Province										
Central	7.5	14.8	8.1	1.03(0.44–2.38)	0.95	3.4	3.0	6.0	1.87 (0.49–7.11)	0.36
Copperbelt	11.1	4.1	5.6	0.48(0.13–1.81)	0.28	0.0	2.0	10	–	
Eastern	10.4	2.9	3.3	0.37(0.14–0.94)**	0.04	4.2	2.1	2	0.53(0.12–2.44)	0.42
Luapula	6.4	8.2	5.6	0.90(0.28–2.85)	0.85	0.0	12	3.7	–	
Lusaka	5.0	8.3	4.0	0.70(0.09–5.55)	0.73	0.0	2.1	1.7	–	
Muchinga	–	–	2.0	–		–	–	2.4	–	
Northern	5.4	3.9	4.5	0.84(0.31–2.28)	0.73	1.1	2.4	6.3	5.96(0.77–46.22)	0.09
N/Western	6.5	3.1	3.7	0.56(0.19–1.64)	0.29	6.6	0.0	2.7	0.39(0.12–1.31)	0.13
Southern	6.9	6.5	4.9	0.68(0.23–2.02)	0.49	3.8	2.6	1.7	0.47(0.08–2.61)	0.39
Western	12.7	8.4	6.1	0.48(0.19–1.21)	0.12	2.4	1.1	3.6	2.02(0.18–22.31)	0.57

The dash (−) represents missing cases

*%* percentage, aOR age – adjusted Odds Ratio and *CI* Confidence Interval

Significant results are in bold (*p* < 0.05)

*P*-values are from the log-binomial regression analysis test for trends. Figures with asterix are significat at *(*p* < 0.10) **(*P* < 0.05) ***(*P* < 0.01)

The data show geographic variation in prevalence trends (Table 2). Significant declines were observed for young women resident in urban Lusaka and rural Eastern province, while there was a non-significant increase in prevalence among young women residing in urban Luapula, North-Western, and Western. Among young men, a strong linear increase was observed in urban Copperbelt, Northern, and Southern provinces. For rural areas, a similar pattern was observed for Central, Copperbelt, and Northern provinces.

## Discussion

HIV prevalence declined among young women aged 15–24 years in the past 12 years in both urban and rural populations, contrasting an increasing trend among young men in urban areas. This contrast by sex differs from previous repeated population-based surveys conducted in the 1990s and early 2000s showing declining trends regardless of sex [14–16]. As a result of the observed trend, the gender disparity in HIV infection narrowed sharply, with the ratio of four and three young women infected for every young man, in urban and rural areas, respectively, in 2001/2 and then being reduced to 1.5 and 1.3 in 2013/14. HIV prevalence among young people has been found to be a good proxy indicator of new infections, and our findings seem not to support the reported UNAIDS estimates of HIV incidence declines in Zambia since 2001 [25].

There was a relative increase in HIV prevalence from 2001/2 to 2013/14 of 38.5 and 97.3% among young men residing in rural and urban areas, respectively, while among young women the corresponding relative declines in rural and urban areas were 41.5 and 29.6%. Mahy et al. (2012), in a study investigating trends in HIV prevalence among young people in generalised epidemics in selected sub- Saharan African countries, including Zambia, between 2000 and 2011, observed that while overall results showed declines in HIV prevalence, further investigations showed differences in trends in prevalence among men and women. Eight of 14 countries had significant declines among young people when women and men were combined. However, nine countries observed significant declines restricted to women only and only two countries revealed significant declines among young men (Cameroon and Tanzania). Estimates from three countries suggested increasing trends for men aged 15–24 [19].

The age-specific pattern of change in HIV prevalence over time among those aged 25 years and older differed somewhat by gender. Prevalence declined substantially in the age groups 25–39 years and was rather stable among those 40 years and/or older among urban men. Among rural men, this pattern of change was less prominent or partly unstable with the exception of a tendency of declines among those aged 35–49 years. The pattern among urban and rural women was closely similar and HIV prevalence tended to increase among those aged 40 years and older. We expected to see a pattern of increase in prevalence in older age groups as a result of the substantial improvement in treatment coverage the past five to 10 years. This gender differential could partly be due to the fact that women are likely to access treatment at an earlier stage of infection as compared to men. Through the Prevention of Mother to Child Transmission (PMTCT) programme, women have had higher access to Antiretroviral Therapy (ART) than men. Health-seeking behaviour is generally also better among women than men, leading to early initiation of ART [26, 27]. Comparisons of HIV prevalence trends by the two age groups (15–19 vs. 20–24) might be difficult to judge due to small numbers.

The first population-based HIV survey conducted in Zambia revealed the HIV prevalence to be 6.9 times higher in women compared to men aged 15–24 years in urban areas [28]. This sharp contrast in transmission has been observed for a long time and found consistently in countries with generalised epidemics [29]. Idele et al. (2014) reported that in Swaziland the HIV prevalence among adolescent girls was five times higher than among adolescent boys and this gap persisted into young adulthood. Similarly in countries such as Botswana, South Africa, and Uganda, the sex disparity is evident among adolescent and young adults [30]. Our observation showing the prevalence ratio in Zambia approaching one raises an urgent research question: What could be the major explanation for this sudden change in the gender difference in HIV prevalence? The differences in the direction of HIV prevalence trends among young women and men may reflect differences in intensity and focus of prevention programmes. In the 1990s, Zambia like many other African countries implemented a multi-sectoral national response with emphasis on an intensive prevention strategy. With the access-to-treatment campaign, much of the preventive focus was gradually weakened or shifted [31]. Women have been prioritised as a target group in prevention programmes, particularly young women, most likely due to the observed sex disparity in HIV infection among young women and men [32]. This was strengthened further with the very successful building up of the prevention of mother-to-child transmission programmes in the past 10 years. Important examples of more recent shifts in prevention policies that might have played a role in explaining gender differentials in transmission trends are the promotion of Voluntary Medical Male Circumcision (VMMC) and the HIV Treatment as Prevention strategy (TasP). TasP raised optimism following studies showing the biological plausibility of antiretroviral therapy to reduce HIV transmission through viral suppression [33]. It is plausible that this may have influenced changes in perceived risk of HIV among young men and consequently their behaviour. Kalichman et al. (2010), in a study investigating the association between sexually transmitted infections and infectiousness beliefs, found that there was an association between believing one is less infectious when viral load is undetectable and being diagnosed with an STI [34]. This finding seems to confirm that infectiousness beliefs play a role in continued HIV transmission risk. Crepaz et al. (2004), in a meta-analytical review, also found that people’s beliefs about HAART and viral load may promote unprotected sex [35].

The participation differentials across survey rounds could have biased the underlying population prevalence estimates. In the two first rounds, the non-participation was about 25% and then reduced to 15% in the most recent round. Observed non-participation was higher among men and mobility may have played a major role. With observed associations between mobility and vulnerability to infection, surveys with substantial non-participation may somewhat underestimate the HIV prevalence among men. However, a study that analysed five population-based surveys found that non-response did not bias national estimates from population-based studies significantly, especially in countries with relatively high prevalence [36, 37]. Barnighausen, using Heckman-type selection models to test and correct for unobserved factors, found evidence of a downward selection bias in existing national HIV prevalence estimates for men in 2007 in Zambia. Based on the assumption that non-participation may be correlated with unobserved personal characteristics, they used a bivariate probit selection model to estimate whether being available for, and consenting to testing is correlated with HIV status [36]. Conversely, the 2013–14 provision of home-based testing seemed to have had a positive impact on participation, with substantially more individuals willing to know their status and consent to HIV testing. We can therefore assume reduced refusal rates among HIV-positive individuals. This might, to some extent, have affected the estimated trend, i.e. assuming reduced underestimation in the final survey round implies that the real prevalence may have declined somewhat more among women than the estimates indicate, and correspondingly that the real increase among young men may be somewhat smaller than what the estimates suggest. The magnitude of these effects is likely to be marginal since the level of improved participation was rather modest.

## Conclusion

In conclusion, the findings of an increase in HIV prevalence among young urban men in the past 12 years, contrasting declining trends among young women in both urban and rural populations, suggests differential effects of prevention efforts. Further, the overall national HIV prevalence decline has thus concealed some striking sex and rural-urban differentials both for trends over time and prevalence levels. This has potential to mislead policies and highlights the need for reconsidering targeted prevention efforts. The evidence in this study suggests that national sub-epidemics must be assessed and considered in a concerted effort to reduce HIV infections.

## Abbreviations

AIDS: Acquired Immunodeficiency Syndrome
ANC: Antenatal Clinic
ART: Antiretroviral Therapy
CDC: Centers for Disease Control and Prevention
DBS: Dried Blood Spot
EA: Enumeration Area
HIV: Human Immunodeficiency Virus
PMTCT: Prevention of Mother to Child Transmission
TasP: HIV Treatment as Prevention strategy
TDRC: Tropical Disease and Research Center
UNAIDS: Joint United Nations Programme on HIV/AIDS
VMMC: Voluntary Medical Male Circumcision
WHO: World Health Organisation
ZDHS: Zambia Demographic and Health Survey
